# Improved Stability of a Stable Crystal Form C of 6S-5-Methyltetrahydrofolate Calcium Salt, Method Development and Validation of an LC–MS/MS Method for Rat Pharmacokinetic Comparison

**DOI:** 10.3390/molecules26196011

**Published:** 2021-10-03

**Authors:** Zenglin Lian, Hong Chen, Kang Liu, Qianghua Jia, Feng Qiu, Yongzhi Cheng

**Affiliations:** 1 Beijing Jinkang Hexin Pharmaceutical Technology Co., Ltd., Beijing 101111, China; zenglinlian@aliyun.com; 2 Beijing Key Lab of TCM Collateral Disease Theory Research, School of Traditional Chinese Medicine, Capital Medical University, Beijing 100069, China; virgo_and_scorpio@163.com; 3 Lianyungang Jinkang Hexin Pharmaceutical Co., Ltd., Lianyungang 222115, China; kang.l@jinkang-chem.com; 4 Chinese Medicine DongFang College, Beijing University, Beijing 100029, China; jiaqianghua@bucmdf.edu.cn

**Keywords:** 6S-5-methyltetrahydrofolate, crystal form C, stability, pharmacokinetics, LC–MS/MS

## Abstract

Folate is a vitamin beneficial for humans that plays an important role in metabolism, but it cannot be well supplemented by food; it is necessary to supplement it in other ways. Based on this consideration, a novel crystal form C of 6S-5-methyltetrahydrofolate calcium salt (MTHF CAC) was obtained. To explore the difference between MTHF CAC and the crystal form Ⅰ of 6S-5-methyltetrahydrofolate calcium salt (MTHF CA) as well as an amorphous product of 6S-5-methyltetrahydrofolate glucosamine salt (MTHF GA), their stability and pharmacokinetic behaviours were compared. The results of high-performance liquid chromatography coupled with ultraviolet detection analysis indicated that MTHF CAC showed a better stability than MTHF CA and MTHF GA. After oral administration of MTHF CAC, MTHF CA, and MTHF GA to male rats, the MTHF concentrations were analysed using a validated liquid chromatography–tandem mass spectrometry, and the pharmacokinetic parameters were compared. The mean residence times (0–t) of MTHF CAC, MTHF CA, and MTHF GA were 3.7 ± 1.9 h, 1.0 ± 0.2 h (*p* < 0.01), and 1.5 ± 0.3 h (*p* < 0.05), respectively. The relative bioavailability of MTHF CAC was calculated to be 351% and 218% compared with MTHF CA and MTHF GA, respectively, which suggests that MTHF CAC can be better absorbed and utilized for a longer period of time.

## 1. Introduction

Folate (vitamin B_9_), a water-soluble vitamin, is beneficial for humans and plays an important role in metabolism, e.g., as an essential cofactor in enzymatic reactions regulating amino acid and nucleic acid metabolism [1,2]. Humans and animals cannot synthesize folate endogenously; thus, it must be obtained by regular intake of folate and related products. However, folate cannot be sufficiently supplemented by diet. The risk of congenital anomalies, such as neural tube defects and congenital heart defects, is increased by folate deficiency. Furthermore, low folate intake has been linked to health disorders, such as Alzheimer’s disease, osteoporosis, and megaloblastic anaemia [3,4,5,6,7]. The recommended daily intake of folate is 240 μg for adults and 400 μg for pregnant women [8,9]. Therefore, it is very crucial to take folate supplementation separately, especially for fertile women.

Folate must be supplemented in a reasonable and correct way, such that human health is not compromised by its inappropriate supplementation. For example, an excessive intake of folate may mask a vitamin B_12_ deficiency, leading to irreversible neuropathy. An acute ingestion of folate may also be lethal [10,11]. In addition, there are several other challenges in folate supplementation, such as low absorption and poor bioavailability [12,13]. Although 6S-5-methyltetrahydrofolate (6S-5-MTHF) was found to be an alternative to folate with potential advantages, such as masking cobalamin deficiency, many studies demonstrated that the oral absorption and bioavailability of 6S-5-MTHF were poor [14,15]. Hence, a crystal form Ⅰ of 6S-5-MTHF calcium salt (MTHF CA) and an amorphous product of 6S-5-MTHF glucosamine salt (MTHF GA, CAS number: 1181972-37-1) were developed as a promising alternative to address this problem [2].

Studies showed that 6S-5-MTHF is not stable and is especially sensitive to oxygen and moisture [16]. Therefore, the storage of 6S-5-MTHF and its related products, such as MTHF CA and MTHF GA, became an urgent problem to be solved. It is necessary to develop stable forms of 6S-5-MTHF. The crystal form of a compound is associated with its pharmacological activity, bioavailability, dissolution, stability, and shelf life, and thus, screening different crystal forms is an important task. Crystal form C of 6S-5-MTHF calcium salt (MTHF CAC), probably a good supplement to folate, was successfully generated in our group using ultrasonic waves to assist crystallization during the formation of the salt. The crystal form C of 6S-5-MTHF calcium salt can be prepared as follows: “First, an equimolar dosage of 0.03 mol (6S)-5-MTHF was added to 325 mL deionized water, and the pH value was adjusted to 7.8 with 10% NaOH solution till (6S)-5-MTHF was fully dissolved. Next, calcium chloride solution (24%, *w*/*w*) was added, and the resulting reaction solution (about 350 mL) was transferred into an ultrasonic reactor with a power density of 0.04 W/mL. The ultrasonic reaction was carried out at 72 °C and maintained for 40 min. The reaction solution was filtered, and the resulting solid was washed with water, ethanol, and acetone, respectively. After drying in vacuum at 25 °C, a dose of 13.5 g white form C of (6S)-5-MTHF calcium salt was obtained.” (Patent No.: US9150982B2) [17]. The X-ray diffraction pattern of MTHF CAC showed diffraction peaks at 2θ angles of 6.3 ± 0.2 and 19.2 ± 0.2 (the X-ray diffraction pattern of MTHF CA showed that the 2θ values of crystal form I are 6.5, 13.3, 16.8 and 20.1). However, the stability and pharmacokinetic behaviour of MTHF CAC still remain unclear.

**Figure 1:**
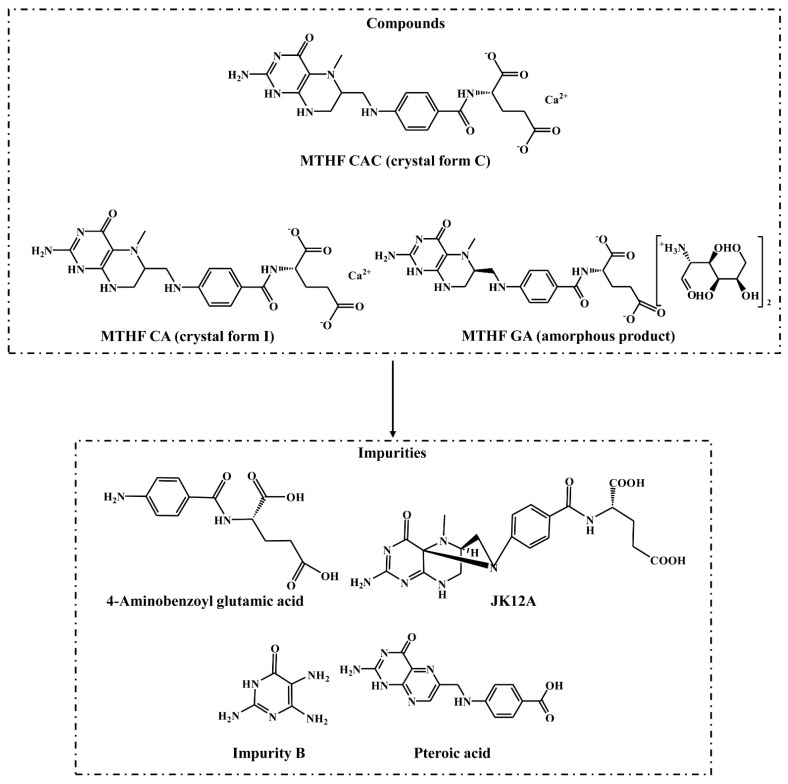
The chemical structures of the crystal form C of 6S-5-methyltetrahydrofolate calcium salt (MTHF CAC), crystal form Ⅰ of 6S-5-methyltetrahydrofolate calcium salt (MTHF CA), amorphous product of 6S-5-methyltetrahydrofolate glucosamine salt (MTHF GA), and four known impurities.

In this study, MTHF CAC is a white crystalline powder containing the crystal form C of 6S-5-MTHF calcium salt, while MTHF CA is a white off-white or yellow beige powder containing of the crystal form Ⅰ of 6S-5-MTHF calcium salt. MTHF GA is a creamy to light brown powder and is an amorphous product of 6S-5-MTHF glucosamine salt. The water content of MTHF CAC and MTHF CA both were 6.0 to 17.0%, and the calcium content of them was 7.0 to 8.5% (on a dry basis), while the water content of MTHF GA was less than 8.0%. The stability of MTHF CAC was first evaluated by comparison with MTHF CA and MTHF GA. The chemicals were modelled as 2D structures using the software ChemDraw Ultra 7.0 (CambridgeSoft, Cambridge, MA, USA) (Figure 1). Furthermore, the pharmacokinetic behaviours of MTHF CAC, MTHF CA, and MTHF GA in Sprague-Dawley rats were investigated after oral administration of an equimolar dosage (0.01 mmol/kg 6S-5-MTHF), providing a comparison of their oral bioavailability and laying the foundation for the follow-up clinical study.

## 2. Results and Discussion

### 2.1. Stability Comparison

**Figure 2:**
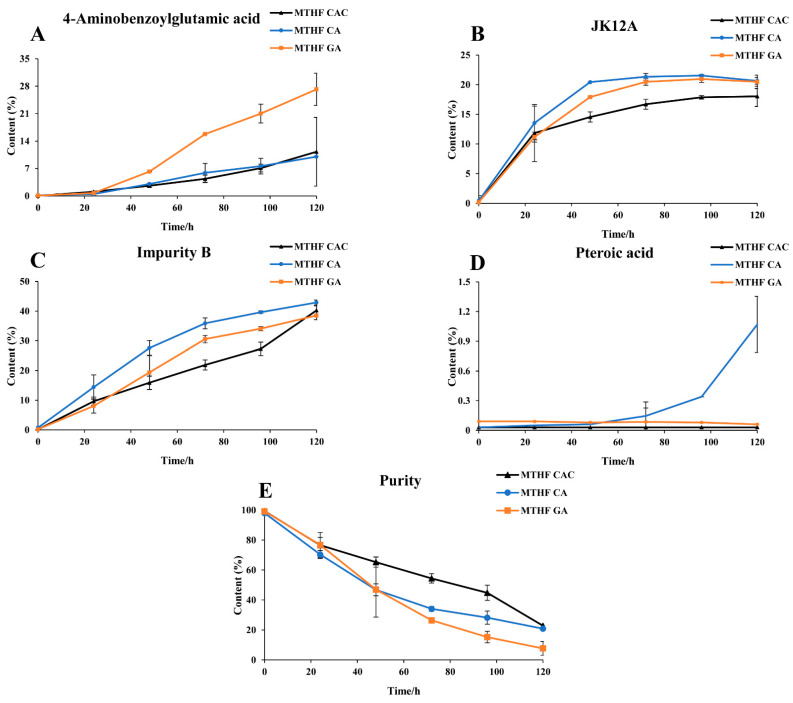
The stability trend comparison of MTHF CAC, MTHF CA, and MTHF GA. Three working solutions containing MTHF CAC, MTHF CA, and MTHF GA were separately prepared. The contents of 4-aminobenzoyl glutamic acid (**A**), JK12A (**B**), impurity B (**C**), pteroic acid (**D**), and 6S-5-methyltetrahydrofolate (6S-5-MTHF) (**E**) were determined at different time points by high-performance liquid chromatography coupled with ultraviolet detection (HPLC–UV).

The stability comparison results are shown in Figure 2. As indicated, the content of 4-aminobenzoyl glutamic acid in MTHF GA at 120 h increased 2.5 times compared with MTHF CAC and MTHF CA (*p* < 0.01), and the degradation rate of 4-aminobenzoyl glutamate in MTHF GA was significantly faster than that in MTHF CAC and MTHF CA (*p* < 0.01). The content of JK12A in MTHF CAC, MTHF CA, and MTHF GA showed a significant change from 0 h to 24 h (*p* < 0.01), and the further increases in MTHF CA and MTHF GA occurred faster than that in MTHF CAC. Finally, the content of JK12A in MTHF CA and MTHF GA was higher than that in MTHF CAC. Among the impurities detected in this experiment, the content of folate impurity B increased the most and showed the greatest change in MTHF CA. During the period of 0–120 h, there was almost no change in the content of pteroic acid in MTHF CAC and MTHF GA, while the content of pteroic acid in MTHF CA increased to 1.08%. As shown in Figure 2, MTHF GA and MTHF CA displayed the same degradation trend during the 0–48 h period, whereas MTHF GA decreased more rapidly from 48 to 72 h. During the periods of 0–24 h and 96–120 h, the degradation of MTHF CAC was significant but tended to be stable at other times. According to their degradation curves, the degradation rate constants (k) of MTHF CAC, MTHF CA, and MTHF GA were calculated to be (7.83 × 10^−3^) ± 0.00, (1.06 × 10^−2^) ± 0.00 and (2.34 × 10^−2^) ± 0.02 s^−1^, respectively. The t_0.9_ values of MTHF CAC, MTHF CA, and MTHF GA were calculated to be 13.4 ± 0.00, 10.2 ± 0.00, and 4.51 ± 0.04 h, respectively. The degradation rate constants (k) and t_0.9_ of MTHF CA and MTHF GA were notably different from those of MTHF CAC (*p* < 0.01). Overall, the degradation of MTHF GA was the fastest, followed by MTHF CA, and MTHF CAC was the most stable (*p* < 0.01).

### 2.2. Pharmacokinetic Comparison

#### 2.2.1. Method Validation

The regression equations and correlation coefficient of 6S-5-MTHF were y = 8.87 × 10^−4^ x + 2.80 × 10^−3^ and 0.9987, respectively. The linear range of standard in rat plasma was 10.0–10,000 ng/mL. The lower limit of quantification (LLOQ) of 6S-5-MTHF was 10.0 ng/mL.

As shown in Table 1, the relative standard deviations (RSDs) for precision were ≤10.9% intra-batch and ≤13.2% inter-batch, and the accuracy was in the range of −7.55% to 12.4%, indicating that both the precision and accuracy met the requirements.

The extraction recoveries of 6S-5-MTHF ranged from 87.4 to 94.8% with no obvious matrix effect in the ratio of the peak area resolved in the post-extraction blank sample and the mobile phase (85.6 to 91.4%) (Table 2). 6S-5-MTHF spiked into rat plasma was stable for 2 h at 25 °C and 24 h at 4 °C, after three freeze-thaw cycles, and for up to 30 days at −80 °C (Table 3). The stability of 6S-5-MTHF for quantitative determination was qualified.

#### 2.2.2. Pharmacokinetic Application

**Figure 3:**
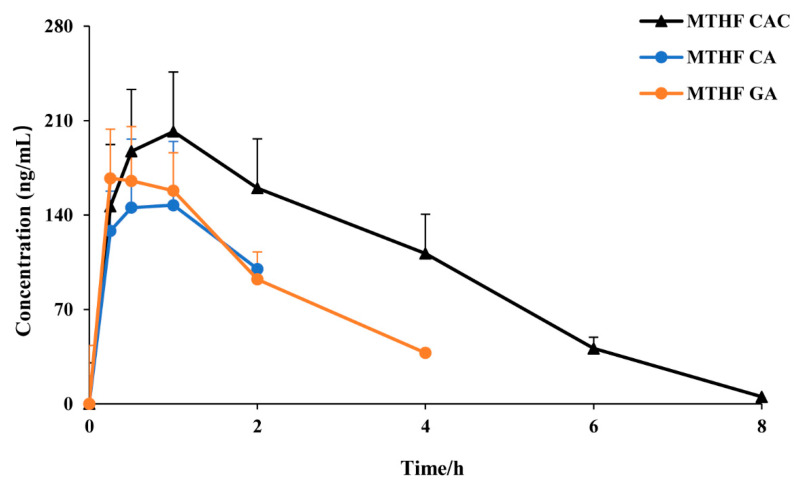
The plasma concentration–time curves of 6S-5-MTHF after oral administration of MTHF CAC, MTHF CA, and MTHF GA to rats (*n* = 6).

To evaluate the trend of the 6S-5-MTHF concentration in rat plasma after administration, a concentration-time curve was plotted (Figure 3). After oral dosing of MTHF CAC, MTHF CA, and MTHF GA, the concentration of 6S-5-MTHF showed a remarkable increase. After oral administration of MTHF CAC, the concentration of 6S-5-MTHF was maintained at a high level in rat plasma for a long period and remained above the base level for 8 h after administration, whereas the MTHF CA-treated and MTHF GA-treated groups had it maintained for approximately 2 and 4 h, respectively.

**Figure 4:**
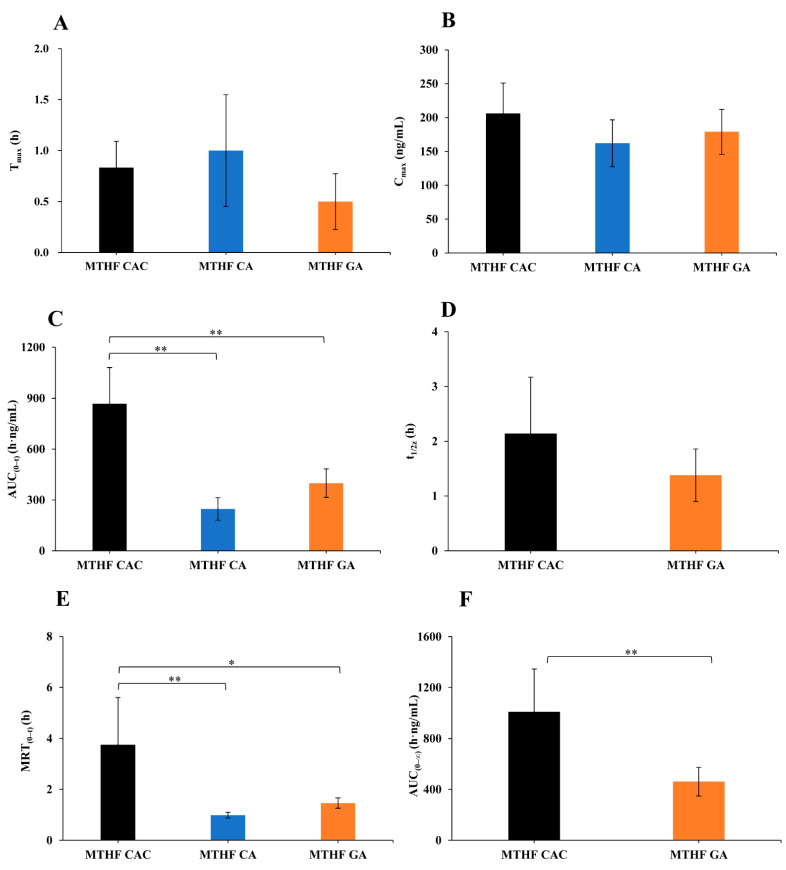
The main pharmacokinetic parameters of 6S-5-MTHF after oral administration of MTHF CAC, MTHF CA, and MTHF GA to male rats at 0.01 mmol/kg (*n* = 6). The average base level was subtracted from the measured concentrations at different time points. (**A**) maximum plasma time (T_max_), (**B**) maximum plasma concentration (C_max_), (**C**) area under the concentration–time curve (AUC_(0–t)_), (**D**) half-life (t_1/2z_), (**E**) mean residence time (MRT_(0–t)_), (**F**) AUC_(0–∞)_. AUC_(0–∞)_ and t_1/2z_ of MTHF CA were not available due to too few effective point data.* *p* < 0.05, ** *p* < 0.01.

The main pharmacokinetic parameters were calculated with the DAS software (Figure 4). To better evaluate the pharmacokinetic parameters of 6S-5-MTHF, the average base level was subtracted from the measured concentrations at different timepoints. After administration of MTHF CAC, MTHF CA, and MTHF GA to male rats at 0.01 mmol/kg, the maximum plasma time (T_max_) values were 0.8 ± 0.3, 1.0 ± 0.5, and 0.5 ± 0.3 h, respectively. According to the disclosure data of patents, MTHF CAC and MTHF CA were prepared by different crystallization methods. MTHF CAC was crystal form C, and MTHF CA was crystal form I. The particle size distribution span of MTHF CAC was 1.8, while that of MTHF CA was 23.9, illustrating that the particle size distribution of MTHF CAC was more uniform than that of MTHF CA. Moreover, the volume weighted mean of MTHF CAC was 10.4, while that of MTHF CA was 28.9, which confirmed that the volume of MTHF CAC was smaller than that of MTHF CA. MTHF GA was an amorphous powder. Thus, the crystal form and crystal size of the compound appeared to have a significant effect on its absorption rate.

After administration of MTHF CAC, MTHF CA, and MTHF GA, the maximum plasma concentration (C_max_) values were 206 ± 40.3, 162 ± 34.6, and 179 ± 33.2 ng/mL, respectively, and the area under the concentration–time curve (0–t) (AUC_(0__–__t)_) values were 868 ± 213, 247 ± 67.0, and 399 ± 83.9 h·ng/mL, respectively. The results suggested that the oral bioavailability of MTHF CAC was superior to that of MTHF CA and MTHF GA. In addition, this study also revealed that the disappearance rate of 6S-5-MTHF in the plasma of rats supplemented with MTHF CAC was strikingly slower than in the case of MTHF GA and MTHF CA supplementation, and the relative bioavailability of MTHF CAC was 351% and 218% in comparison with MTHF CA and MTHF GA, respectively.

### 2.3. Discussion

#### 2.3.1. Sample Preparation

According to the results of the stability comparison, attention should be given to the degradation of 6S-5-MTHF in the analysis process. The effects of various stabilizers, such as dithiothreitol (DTT) and vitamin C (Vc), on the degradation of folate were thus compared. The experimental results revealed that degradation could be avoided effectively when DDT and Vc were used in combination at every step of sample preparation.

#### 2.3.2. Mass Spectrometry and Chromatography

**Figure 5:**
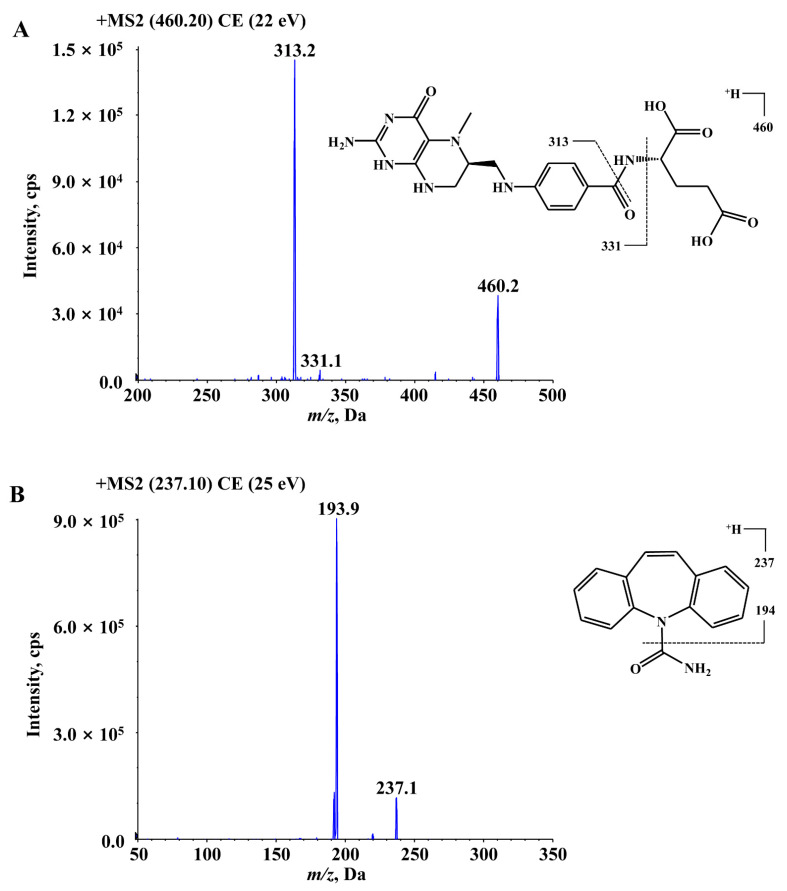
The MS2 product ions spectra and fragmentation patterns of 6S-5-MTHF (**A**) and carbamazepine (IS) (**B**).

In this study, the development and validation of a sensitive and specific liquid chromatography–tandem mass spectrometry (LC–MS/MS) assay for the determination of 6S-5-MTHF in rat plasma was described. The MS2 product ion spectra and fragmentation patterns of 6S-5-MTHF and carbamazepine (internal standard, IS) are shown in Figure 5. In the full-scan Q1 mass spectrum of 6S-5-MTHF, the protonated ion [M + H]^+^ (*m*/*z* 460.2) was the most abundant and sufficiently stable, and was used to screen the product ions for identification and quantification. As shown in Figure 5, the product ions with the strongest responses in 6S-5-MTHF were two ions at *m*/*z* 333.1 and *m*/*z* 313.2. The ion at *m*/*z* 313.2 may be formed by the fragmentation of the acyl amide bond, and its signal was stronger than that of the ion at *m*/*z* 333.1. Hence, the ion at *m*/*z* 313.2 was used for quantification, whereas the ion at *m*/*z* 333.1 was used for identification. The mass spectrometry parameters of 6S-5-MTHF, such as ion spray voltage, declustering potential (DP), curtain gas pressure (CUR), collision gas (CAD), ion source gas 1 (GS1), ion source gas 2 (GS2), capillary temperature, and collision energy (CE), were optimized. The MS parameters for the carbamazepine transition *m*/*z* 237.1 → 193.9 were optimized in the same way as those for 6S-5-MTHF.

**Figure 6:**
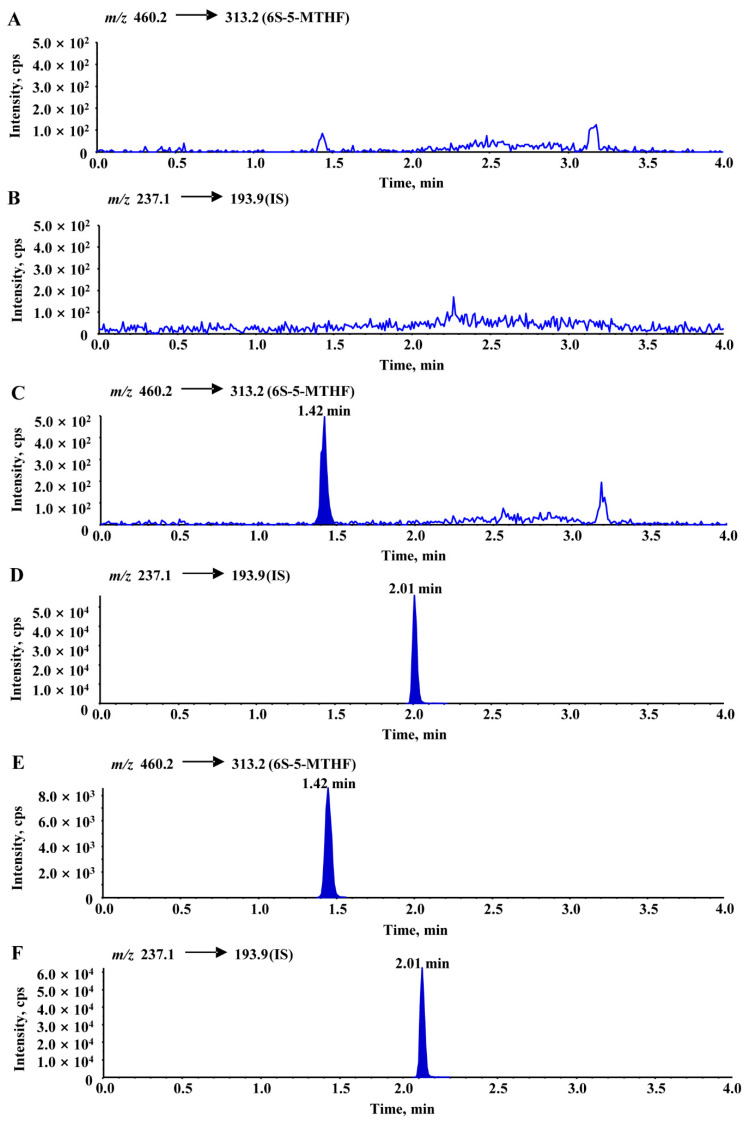
Typical chromatograms of blank rat plasma ((**A**): 6S-5-MTHF, (**B**): IS), blank rat plasma spiked with 6S-5-MTHF (10 ng/mL, LLOQ) and IS ((**C**): 6S-5-MTHF, (**D**): IS), an unknown rat plasma sample collected at 15 min after oral administration ((**E**): 6S-5-MTHF, (**F**): IS). In the analyses, the average blank interference was calculated from multiple blank rat plasma samples and was subtracted from the measured results.

Representative multiple reaction monitoring (MRM) chromatograms of 6S-5-MTHF and carbamazepine obtained from blank rat plasma, blank plasma spiked with 6S-5-MTHF and carbamazepine, and actual unknown plasma samples obtained from rats after oral administration of an equimolar dosage (0.01 mmol/kg 6S-5-MTHF) are shown in Figure 6. Under the above chromatographic conditions, there were no obviously endogenous disturbances observed during the retention times of 6S-5-MTHF and carbamazepine, and the chromatographic peaks of 6S-5-MTHF and carbamazepine were separated from each other. Only 4.0 min was used for the chromatographic running time of each rat plasma sample.

The presence of folate in rats caused the blank to interfere with a response of approximately 100 cps. In the subsequent analyses, the average blank interference was calculated from multiple blank rat plasma samples and was subtracted from the measured results.

#### 2.3.3. Pharmacokinetics

It was indicated that MTHF CAC showed better stability than MTHF CA and MTHF GA in vitro and thus in gastrointestinal tract and showed better absorption in rats mostly due to better release in vivo. Moreover, the crystal form of MTHF CAC (crystal form C) is different from that of MTHF CA (crystal form Ⅰ) and MTHF GA (an amorphous product), so the solubility of MTHF CAC might have been also different to some extent. However, the biofilm permeability of MTHF CAC, MTHF CA, and MTHF GA might be the same because of their same chemical structure. In this study, the relative bioavailability of MTHF CAC was calculated to be 351% and 218% compared with MTHF CA and MTHF GA, respectively, which suggested that MTHF CAC could be better absorbed and utilized for a longer period of time. Combined with the above experimental results, this suggests the conclusion that MTHF CAC may have better absorption and can be utilized and functional for a longer time. It may be caused by the differences in the crystal form and chemical properties.

Significant differences in T_max_ or C_max_ values were not observed in MTHF CAC. However, the results demonstrated that MTHF CAC could be continuously absorbed in rats over a longer period of time and its elimination was slower than that of MTHF CA and MTHF GA, leading to higher bioavailability, presumably due to the better release of MTHF CAC in vivo.

## 3. Materials and Methods

### 3.1. Chemicals and Materials

The reference standards of 6S-5-MTHF and four known impurities (4-aminobenzoyl glutamic acid, JK12A, impurity B, and pteroic acid, Figure 1) were provided by Lianyungang Jinkang Hexin Pharmaceutical Co. Ltd. (Lianyungang, Jiangsu, China). Three 6S-5-MTHF products MTHF CAC (purity 99.9%, batch No. 150727), MTHF CA (purity 98.5%, batch No. LMCG046901), and MTHF GA (purity 99.1%, batch No. F5P4002) were provided by Lianyungang Jinkang Hexin Pharmaceutical Co. Ltd. (Lianyungang, Jiangsu, China). HPLC-grade methanol and formic acid were purchased from Honeywell (Charlotte, NC, USA) and Dikma (Beijing, China), respectively. EDTA dipotassium salt (EDTA-K_2_) was supplied by Beijing Sinopharm Chemical Reagent Co., Ltd. (Beijing, China). Water was distilled and deionized using a Milli-Q Reagent water system (Millipore, MA, USA). Blank rat plasma (drug-free and anticoagulated with EDTA-K_2_) was prepared in our laboratory. All other chemicals were classified as analytical grade.

### 3.2. Stability Comparison

Three working solutions containing MTHF CAC, MTHF CA, or MTHF GA were separately prepared, using distilled deionized water, with a concentration of 0.50 mg/mL calculated for the form 6S-5-MTHF. These three solutions were then sealed using a centrifuge tube and placed at room temperature (25 °C). Four known impurities (4-aminobenzoyl glutamic acid, JK12A, impurity B, and pteroic acid [18]) and 6S-5-MTHF were determined at 0, 24, 48, 72, 96 and 120 h by a HPLC–UV technique [19] developed in our laboratory.

From the degradation curves in Figure 2, it was deduced that the degradation behaviour of MTHF followed the first-order kinetics, and then the degradation rate constants (k) of MTHF CAC, MTHF CA, and MTHF GA were calculated according to the degradation curves in Figure 2. The corresponding t_0.9_ values were calculated using the following equation: t_0.9_ = 0.1054/k.

In this study, the significances of differences in the data were analysed using the statistical software package IBM SPSS 23.0 (IBM, Chicago, IL, USA). All the results were analysed by one-way analysis of variance (ANOVA). A *p*-value of less than 0.05 and 0.01 implied significance and high significance, respectively.

### 3.3. Pharmacokinetic Comparison

#### 3.3.1. Method Validation

The determination of 6S-5-MTHF in rat plasma was conducted according to international guidelines [20,21,22,23,24].

The chromatograms of blank plasma samples collected from six different rats were compared with those of plasma samples spiked with 6S-5-MTHF and IS to evaluate the selectivity of the present LC–MS/MS method. The slope, intercept, and correlation coefficient (r^2^) of each linear regression equation were determined using the least-squares linear regression method (1/x^2^ weighting).

Four concentration levels of quality control (QC) samples (LLOQ, low, medium, and high levels) were prepared in six replicates to evaluate the precision and accuracy of the measurements. The RSD was used to express precision, and the relative error (RE) was used to express accuracy. The precision and accuracy of the standards at the lowest concentration (10.0 ng/mL) were also assessed, representing the LLOQ for the assay.

The absolute recovery was calculated according to the comparison of the responses of 6S-5-MTHF in QC samples and those 6S-5-MTHF-spiked in post-extraction blank rat plasma at equivalent concentrations. The matrix effect was assessed by contrasting the responses from spiked, blank rat plasma samples after extraction with those from the mobile phase mixed with LLOQ, and low, medium, and high concentrations of 6S-5-MTHF.

The QC samples (stored at room temperature for 2 h, in autosampler (4 °C) for 24 h, after three freeze-thaw cycles, and long-term stored (−80 °C) for 30 days) were measured to evaluate the stability of 6S-5-MTHF in rat plasma.

#### 3.3.2. Animals

Eighteen healthy male Sprague–Dawley rats (weight 210 ± 30 g, 6–8 weeks of age) were purchased from Beijing Vital River Laboratories Co., Ltd. (Beijing, China). The protocol was approved by the Animal Ethics Committee of Capital Medical University (Beijing, China) with no. AEEI-2015-180 and followed the Guide for Care and Use of Laboratory Animals [25]. Animals were housed in an environmentally controlled room at 22 ± 3 °C with a relative humidity of 60–100% with standard laboratory food. All animals were fed for at least five days to acclimate before being used and were fasted for 16 h prior to the study. Water was freely available during experiments.

#### 3.3.3. Chromatography and Mass Spectrometry Conditions

An Agilent 1200 series HPLC system (Agilent Technologies, CA, USA) was used for liquid chromatographic analysis. Chromatographic separation was performed with a Grace Altima HP C_18_ column (50 mm × 2.1 mm, 5 μm) at 4 °C. The samples were separated with solvent A (methanol/water/acetic acid = 10:90:0.1, *v*/*v*/*v*) and solvent B (methanol/acetic acid = 100:0.1, *v*/*v*) at a flow rate of 0.4 mL/min for 4.00 min. The optimum conditions were achieved according to the following flow program: 0.00–0.10 min (0% B), 0.10–1.00 min (0–90% B), 1.00–2.00 min (90% B), 2.00–2.10 min (90–0% B), and 2.10–4.00 min (0% B). The injection volume was 5 μL.

Mass spectrometry detection was conducted using an API-4000 mass spectrometer (Applied Biosystems/MDS Sciex, Concord, ON, Canada) equipped with a turbo ion spray source in positive ion mode. MRM mode was used to determine the samples. The MS parameters of 6S-5-MTHF and carbamazepine (internal standard, IS) are shown in Table 4, and the experimental data were recorded and analysed by the Analyst software (version 1.6.1, Applied Biosystems/MDS Sciex, Concord, ON, Canada).

#### 3.3.4. Sample Preparation

The rat plasma was placed at room temperature for approximately 30 min to thaw and then vortexed for 30 s. Aliquots of 50 μL plasma samples were added into centrifuge tubes, then 5 μL of methanol and 300 μL of IS solution were added (simultaneously containing 40 ng/mL carbamazepine, 1 mg/mL DTT, and 1 mg/mL Vc in methanol). After vortexing for 1 min and then centrifuging at 12,000 × *g* for 10 min, 20 μL aliquots of the supernatant were mixed with 80 µL of a stabilizing solution (DTT and Vc mixed aqueous solution, both 1 mg/mL) before transfer to liquid vials for subsequent analysis.

#### 3.3.5. Preparation of Standard and Quality Control Samples

Stock solutions of 6S-5-MTHF and carbamazepine (both 1.00 mg/mL) were prepared separately in dimethyl sulfoxide (DMSO). Two primary stock solutions of 6S-5-MTHF were prepared to ensure weighing precision (their concentrations agreed within 5%); one was used for calibration-curve standards and the other was used for QC samples. The stock solution of 6S-5-MTHF was further diluted with methanol to obtain working standard solutions with final concentrations of 10.0, 20.0, 50.0, 200, 500, 1000, 5000, and 10,000 ng/mL for the preparation of calibration curves. QC samples were prepared at 6S-5-MTHF concentrations of 10.0, 20.0, 500, and 8000 ng/mL using the same process as for the calibration samples. IS solution (containing 40 ng/mL carbamazepine, 1 mg/mL DTT, and 1 mg/mL Vc in methanol) was also prepared by diluting the carbamazepine stock solution with methanol. All standard solutions were stored at 4 °C. Aliquots of 50 μL of drug-free rat plasma sample were mixed with 300 μL of IS solution (simultaneously containing 40 ng/mL carbamazepine, 1 mg/mL DTT, and 1 mg/mL Vc in methanol) and 5 μL of serial standard solutions containing 6S-5-MTHF for the preparation of the calibration standards at concentrations ranging from 10.0 to 10,000 ng/mL. Control rat plasma was added in bulk at appropriate concentrations to prepare QC samples and then divided into small aliquots (approximately 80 μL) in different tubes. These samples and the real rat plasma samples were stored under the same conditions and subjected to the same pre-treatment procedure.

#### 3.3.6. Rat Pharmacokinetics

Three days before the rat pharmacokinetic experiment, polyethylene cannulas were implanted into the femoral veins after the rats had been anaesthetized with pentobarbital (50 mg/kg, intravenous). The cannulas were exposed at the back of the neck and filled with EDTA-K_2_ anticoagulant saline (20 units/mL). Before the experiment, rats were fasted for 16 h, while water was always available.

Oral dosing solutions of MTHF CAC, MTHF CA, and MTHF GA (0.459 mg/mL calculated in the form of 6S-5-MTHF) were separately prepared by dissolving appropriate amounts of three compounds in purified water with the addition of Vc (0.8 mg/mL) as an antioxidant agent. The concentrations of 6S-5-MTHF in these three oral dosing solutions were confirmed by the LC–MS/MS method above. The oral dose of 6S-5-MTHF was 0.01 mmol/kg [8,9], which corresponded to an oral dose of 10.0 mL/kg in this experiment. After oral administration, 0.20 mL blood samples were collected in EDTA-K_2_ anticoagulant polyethylene tubes at predetermined time intervals (pre-dose, 0.25, 0.50, 1.00, 2.00, 4.00, 6.00, and 8.00 h). The EDTA-K_2_ anticoagulant blood was centrifuged at 12,000× *g* at room temperature for 5 min to obtain plasma and then stored at −80 °C before analysis. The health status of the animals within 2 h after dosing was observed, and then the animals were observed and recorded at each timepoint until the final samples were collected.

The pharmacokinetic parameters, including half-life (t_1/2z_), T_max_, C_max_, AUC_(0–t)_ and AUC_(0–∞)_, and mean residence time (MRT_(0–t)_) of 6S-5-MTHF, were analysed by the non-compartmental method using DAS, version 2.0 (Chinese Pharmacological Society, Beijing, China). All the data were expressed as the arithmetic mean ± standard deviation (SD).

#### 3.3.7. Statistical Analysis

In this study, significant differences in the data were analysed using the statistical software package IBM SPSS 23.0 (IBM, Chicago, IL, USA). All the results were analysed by one-way ANOVA. A *p*-value of less than 0.05 and 0.01 implied significance and high significance, respectively.

## 4. Conclusions

MTHF CAC showed greater stability than MTHF CA and MTHF GA. In addition, MTHF CAC was well absorbed and better utilized in SD rats in contrast to MTHF CA and MTHF GA. The results indicated that MTHF CAC might be a good supplement to folate.

## Figures and Tables

**Table 1:** Intra-batch and inter-batch precisions and accuracies of 6S-5-MTHF in rat plasma determined by liquid chromatography–tandem mass spectrometry (LC–MS/MS) (*n* = 6).

Spiked Concentration (ng/mL)	Precision (%, RSD)		Accuracy (%, RE)
	Intra-Batch	Inter-Batch	
10.0	9.24	13.1	12.4
20.0	10.8	11.5	8.03
500	7.33	6.18	−7.55
8000	5.82	8.36	9.27

**Table 2:** Matrix effects and recoveries of 6S-5-MTHF in rat plasma determined by LC–MS/MS (*n* = 6).

Spiked Concentration (ng/mL)	Recovery (%)	RSD (%)	Matrix Effect (%)	RSD (%)
10.0	87.4	10.4	89.8	14.1
20.0	89.1	9.48	85.6	10.2
500	91.7	12.2	88.7	9.38
8000	94.8	4.38	91.4	6.48

**Table 3:** Stability data of 6S-5-MTHF under different storage conditions determined by LC–MS/MS (*n* = 6).

Storage Condition	Concentration (ng/mL)		RSD (%)	Accuracy (%, RE)
	Spiked	Measured		
Room temperature (2 h at 25 °C)	20.0	18.3	5.89	−8.50
	500	523	7.11	4.60
	8000	8220	8.95	2.75
Autosampler for 24 h (4 °C)	20.0	21.2	7.43	6.00
	500	517	8.63	3.40
	8000	8310	10.1	3.88
Three freeze/thaw cycles	20.0	17.6	12.6	−12.0
	500	477	7.53	−4.60
	8000	7660	13.9	−4.25
Long-term (30 days at −80 °C)	20.0	21.2	11.6	6.00
	500	465	12.6	−7.00
	8000	7540	10.9	−5.75

**Table 4:** MS parameters of 6S-5-MTHF and carbamazepine determined by LC–MS/MS.

MS Parameters	6S-5-MTHF for Quantification	6S-5-MTHF for Identification	Carbamazepine (IS)
Q1 (*m*/*z*)	460.2	460.2	237.1
Q3 (*m*/*z*)	313.2	331.1	193.9
Ion Spray (V)	5500	5500	5500
DP (V)	80	80	80
CUR (psi)	25	25	25
GS 1 (psi)	50	50	50
GS 2 (psi)	50	50	50
CAD	4	4	4
Capillary Temp. (°C)	450	450	450
CE (eV)	25	18	28
CXP (V)	9	9	7

## Data Availability

The data presented in this study are available in the article.

## Sample Availability

Samples of the materials are available from the authors.

## References

[1] Sobczyńska-Malefora A., Harrington D.J. (2018). Laboratory assessment of folate (vitamin B9) status. J. Clin. Pathol..

[2] Miraglia N., Agostinetto M., Bianchi D., Valoti E. (2016). Enhanced oral bioavailability of a novel folate salt: Comparison with folic acid and a calcium folate salt in a pharmacokinetic study in rats. Minerva Ginecol..

[3] Czeizel A.E., Dudás I., Vereczkey A., Bánhidy F. (2013). Folate deficiency and folic acid supplementation: The prevention of neural-tube defects and congenital heart defects. Nutrients.

[4] Greene N.D., Leung K.Y., Copp A.J. (2017). Inositol, neural tube closure and the prevention of neural tube defects. Birth Defects Res..

[5] Wang Y., Jin Y., Wang Y., Li L., Liao Y.H., Zhang Y., Yu D. (2019). The effect of folic acid in patients with cardiovascular disease: A systematic review and meta-analysis. Medicine.

[6] Sid V., Siow Y.L., O K. (2017). Role of folate in nonalcoholic fatty liver disease. Can. J. Physiol. Pharmacol..

[7] Guilland J.C., Aimone-Gastin I. (2013). Vitamine B9. Rev. Prat..

[8] Nagao T., Hirokawa M. (2017). Diagnosis and treatment of macrocytic anemias in adults. J. Gen. Fam. Med..

[9] Gebremichael T.G., Welesamuel T.G. (2020). Adherence to iron-folic acid supplement and associated factors among antenatal care attending pregnant mothers in governmental health institutions of Adwa town, Tigray, Ethiopia: Cross-sectional study. PLoS ONE.

[10] Devnath G.P., Kumaran S., Rajiv R., Shaha K.K., Nagaraj A. (2017). Fatal Folic Acid Toxicity in Humans. J. Forensic Sci..

[11] Jungert A., Quack Lötscher K., Rohrmann S. (2020). Vitamin Substitution Beyond Childhood-Requirements and Risks. Deutsches Ärzteblatt international..

[12] Pietrzik K., Bailey L., Shane B. (2010). Folic acid and L-5-methyltetrahydrofolate: Comparison of clinical pharmacokinetics and pharmacodynamics. Clin. Pharmacokinet..

[13] Pillay D., Wham C., Moyes S., Muru-Lanning M., Teh R., Kerse N. (2018). Intakes, Adequacy, and Biomarker Status of Iron, Folate, and Vitamin B12 in Māori and Non-Māori Octogenarians: Life and Living in Advanced Age: A Cohort Study in New Zealand (LiLACS NZ). Nutrients.

[14] Scaglione F., Panzavolta G. (2014). Folate, folic acid and 5-methyltetrahydrofolate are not the same thing. Xenobiotica..

[15] Henderson A.M., Aleliunas R.E., Loh S.P., Khor G.L., Harvey-Leeson S., Glier M.B., Kitts D.D., Green T.J., Devlin A.M. (2018). l-5-Methyltetrahydrofolate Supplementation Increases Blood Folate Concentrations to a Greater Extent than Folic Acid Supplementation in Malaysian Women. J. Nutr..

[16] Fitzhugh A.L. (1993). Stereoelectronic effects in the autoxidative destruction of reduced folate derivatives. Adv. Exp. Med. Biol..

[17] Wang Z., Cheng Y., Huang H., Li H. (2015). Crystal Form Of (6s)-5-Methyltetrahydrofolate Salt and Method For Preparing Same. U.S. Patent.

[18] Pierce M., Kahn J.N., Chiou J., Tumer N.E. (2011). Development of a quantitative RT-PCR assay to examine the kinetics of ribosome depurination by ribosome inactivating proteins using Saccharomyces cerevisiae as a model. RNA.

[19] Akhtar M.J., Khan M.A., Ahmad I. (1997). High performance liquid chromatographic determination of folic acid and its photodegradation products in the presence of riboflavin. J. Pharm. Biomed. Anal..

[20] US Food and Drug Administration (2018). Guidance for Industry: Bioanalytical Method Validation.

[21] European Medicines Agency (2011). Guideline on Bioanalytical Method Validation.

[22] Fu C.Q., Yu P., Wang M.Y., Qiu F. (2020). Phytochemical analysis and geographic assessment of flavonoids, coumarins and sesquiterpenes in Artemisia annua L. based on HPLC-DAD quantification and LC-ESI-QTOF-MS/MS confirmation. Food Chem..

[23] Zhang P., Ma H., Lin X.L., Qiu F. (2020). Simultaneous quantification and rat pharmacokinetics of formononetin-7-O-β-d-glucoside and its metabolite formononetin by high-performance liquid chromatography-tandem mass spectrometry. J. Sep. Sci..

[24] Fu C.Q., Shi H.N., Chen H., Zhang K.Y., Wang M.Y., Qiu F. (2020). Oral Bioavailability Comparison of Artemisinin, Deoxyartemisinin, and 10-Deoxoartemisinin Based on Computer Simulations and Pharmacokinetics in Rats. ACS Omega..

[25] National Research Council (2010). Guide for the Care and Use of Laboratory Animals.

